# Retinoic acid and liver X receptor agonist synergistically inhibit HIV infection in CD4+ T cells by up-regulating ABCA1-mediated cholesterol efflux

**DOI:** 10.1186/1476-511X-11-69

**Published:** 2012-07-09

**Authors:** Hong Jiang, Yunden Badralmaa, Jun Yang, Richard Lempicki, Allison Hazen, Ven Natarajan

**Affiliations:** 1 Laboratory of Molecular Cell Biology, SAIC-Frederick, Inc, Frederick National Laboratory, Frederick, MD, USA, 21702; 2 Laboratory of Laboratory of Immunopathogenesis and Bioinformatics, SAIC-Frederick, Inc, Frederick, MD, USA, 21702

**Keywords:** ABCA1, ATRA, retinoic acid, TO-901317, RAR, RXR, LXR, cholesterol efflux, HIV-1, CD4+ T cells

## Abstract

**Background:**

Retinoic acids regulate the reverse cholesterol transport by inducing the ATP binding cassette transporter A1 (ABCA1) dependent cholesterol efflux in macrophages, neuronal as well as intestine cells. In the present study, we aim to test the effect of all trans retinoic acid (ATRA) on ABCA1 expression in human CD4+ T cells and the involvement of cholesterol in ATRA mediated anti-HIV effect.

**Results:**

Treatment with ATRA dramatically up-regulated ABCA1 expression in CD4+ T cells in a time and dose dependent manner. The expression of ABCA1 paralleled with increased ABCA1-dependent cholesterol efflux. This induction was dependent on T cell receptor (TCR) signaling and ATRA failed to induce ABCA1 expression in resting T cells. Moreover, ATRA and liver X receptor (LXR) agonist-TO-901317 together had synergistic effect on ABCA1 expression as well as cholesterol efflux. Increased ABCA1 expression was associated with lower cellular cholesterol staining. Cells treated with either ATRA or TO-901317 were less vulnerable to HIV-1 infection. Combination of retinoic acid and TO-901317 further inhibited HIV-1 entry and their inhibitory effects could be reversed by cholesterol replenishment.

**Methods:**

ABCA1 RNA and protein were determined by real-time PCR and immuno blot methods in cells treated with ATRA. Cholesterol efflux rate was measured in cells treated with ATRA and TO-901317.

**Conclusions:**

ATRA up-regulates ABCA1 expression and cholesterol efflux in CD4+ T cells and combination of ATRA and liver X receptor **(**LXR) agonist further enhanced these effects. Increased cholesterol efflux contributed to reduced HIV-1 entry, suggesting that anti-HIV effect of ATRA is mediated through ABCA1.

## Background

Lipid components of the cell membrane are important for normal cell function. Cholesterol is one of the most important regulators of lipid organization. It is also the major component of lipid rafts, which are the centers for assembling of signaling molecules and membrane protein trafficking [1]. Lipid rafts are also believed to be sites for HIV-1 entry, assembly and budding [2-5]. Cholesterol on both viral and cellular membrane is required for successful HIV-1 infection. Down-regulation of cholesterol from HIV-1 target cells dramatically inhibited both HIV-1 entry and virus particle production [6,7]. Removal of the cholesterol from HIV-1 with cholesterol extraction reagent β-cyclodextrin resulted in a dose-dependent inactivation of the virus [8].

Cellular cholesterol is maintained in a narrow range by cholesterol up-take and efflux. Accumulation of cholesterol can have profound effects on cellular functions, which can cause serious diseases, like atherosclerosis [9]. ABCA1, a member of the ATP-binding cassette transporter protein family (ABCs), plays an essential role in controlling the cellular cholesterol level by mediating the cellular free cholesterol efflux to lipid-free apolipoprotein A1 (apo-A1) [10]. ABCA1 is a ubiquitously expressed plasma membrane protein. ABCA1 mutation and deficiency is associated with increased tissue and cellular cholesterol, atherosclerosis and Tangier disease [11-15].

Regulations of ABCA1 and lipid efflux have been studied extensively in macrophages. LXR and LXR ligand oxysterol play a major role in ABCA1 induction and cholesterol efflux in macrophages [16-18]. Retinoic acids by binding to retinoic acid receptor (RAR) and retinoid X receptor (RXR) are also known to induce ABCA1 expression in macrophages [16,19,20]. Retinoic acid is involved in multiple T cell functions and very little is known about the regulation of ABCA1 in T cells. In this study, we investigated the regulation of ABCA1 expression and cholesterol efflux in T cells by ATRA. Our results demonstrated that ATRA specifically up-regulated ABCA1 but not ABCA3 or ABCG1 expression. ABCA1 mediated free cholesterol efflux, which contributed to significant reduction of HIV-1 entry into T cells. Furthermore, ATRA and TO-901317, an LXR agonist, functioned synergistically to further enhance ABCA1 expression and inhibit HIV-1 infection in T cells.

## Results

### Up regulation of ABCA1 in CD4+ T cells by ATRA

**Figure 1:**
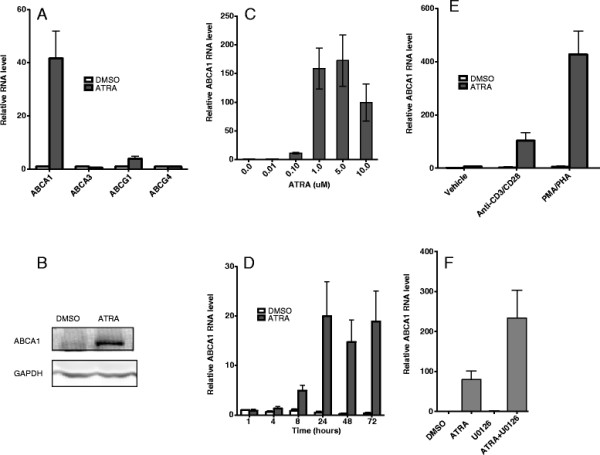
**ATRA induces ABCA1 expression in human primary CD4+ T cells.A**) Human CD4+ T cells isolated from healthy donors were primed with anti-CD3 and anti-CD28 antibodies and treated with DMSO or 1 μM of ATRA for 3 days. RNA isolation, reverse transcription, and real time PCR were done as described in Methods. ABCA1 RNA expression was normalized to GAPDH RNA expression, and relative RNA fold changes compared to that from DMSO treatment were plotted. **B**) ABCA1 proteins were detected by Western blot with antibody specific to human ABCA1 as described. GAPDH was used as a loading control. **C**) ABCA1 RNA expression in response to treatment with different concentrations of ATRA. **D**) ABCA1 RNA expression in response to treatment with ATRA for various lengths of times. **E**) CD4+ T cells were activated with anti-CD3 and anti-CD28 antibody or PMA and PHA and were also treated with either DMSO or ATRA. ABCA1 RNA level was analyzed by real-time PCR. **F**) Anti-CD3/CD28 antibody primed CD4+ T cells were treated with DMSO or 10 μM of U0126 for 2 hours and then treated with or without ATRA. ABCA1 RNA level was quantitated by real-time PCR. Results are representative of three experiments with cells from three different donors.

Retinoic acids have been shown to influence the function of T cells while its effect in T cells has not been fully understood [21,22]. PMA and PHA or antibodies against CD3 and CD28 are used to activate T cells in vitro. PHA and PMA activate T cells by binding to cell surface receptor including TCR and activating protein kinase C respectively. Bindings of antibodies against CD3 and CD28 to corresponding receptor activate T cells by mimicking the intracellular signals generated by ligation of TCR-CD3 [23]. To assess the effect of ATRA on gene expressions, we treated anti-CD3/CD28 antibody primed CD4+ T cells from healthy donors with or without ATRA, and RNAs from these cells were isolated and used for gene array analysis and identified ABCA1 as one of the most up regulated gene by ATRA treatment. To confirm this result, changes of ABCA1 mRNA levels in response to ATRA treatment was assessed by quantitative real-time PCR following reverse transcription. Consistent with gene array results, ABCA1 mRNA was dramatically up regulated by ATRA treatment (Figure 1A). Since ABCA1 RNA stability was not affected in response to ATRA treatment (data not shown), the up-regulation of ABCA1 is at the transcription level. This is consistent with previous findings seen in macrophages [20]. The effect of ATRA on ABCA1 protein expression was also analyzed by western blot. As shown in Figure 1B, the basal expression of ABCA1 protein is barely detectable in primary human CD4+ T cells. In response to the stimulation with ATRA, ABCA1 protein level significantly increased, which parallels with the induction of its mRNA. The induction of ABCA1 expression was both time- and dose-dependent. ATRA up-regulated ABCA1 RNA by 11 fold at 0.1 μM, and at concentrations of 1 μM and 5 μM could induce ABCA1 RNA expression over 100 times (Figure 1C). As early as 4 hours after ATRA treatment, the expression of ABCA1 mRNA increased by almost 3 times and by 24 hrs of treatment, the stimulation of ABCA1 expression reached maximum (Figure 1D).

### ABCA1 induction by ATRA is dependent on TCR signaling

ATRA has only marginal effect on ABCA1 expression in resting CD4+ T cells. Upon T cell activation with anti-CD3 and CD28 antibodies, the expression of ABCA1 increased 100 folds in response to ATRA treatment. PMA/PHA treatment together with ATRA increased the ABCA1 expression by 400 folds (Figure 1E). Whereas, without ATRA, T cell activation alone had little effect. These results indicate that both ATRA and TCR signaling are required for ABCA1 expression and TCR signaling is essential for ATRA effect on ABCA1 up regulation. During T cell activation, MAP kinase pathways including ERK pathway are affected [24]. ERK signaling pathway has been shown to play a role in ABCA1 mRNA and protein stability in macrophages [25]. When different MAP kinase inhibitors were tested on ABCA1 mRNA levels, none of the inhibitors by themselves had any effect on ABCA1 mRNA expression (data not shown). However, ERK inhibitor along with ATRA had significant stimulatory effect on ABCA1 (Figure 1F). The mechanism of up regulation of ABCA1 mRNA in CD4+ T cells by ERK inhibitor is not known yet but it could stabilize newly synthesized ABCA1 mRNA and protein as in macrophages [25].

ABCA1 is a ubiquitously expressed plasma membrane protein. It belongs to a family of proteins called ATP-binding cassette transporter (ABCs). There are 49 human ABC proteins. They are classified into seven subfamilies, from A to G based on the similarity in their gene structure, sequence or phylogenesis. Besides ABCA1, ABCG1 is also capable of mediating cholesterol efflux [10]. Also, ABCA1 and ABCG1 appear to share a similar mechanism of regulation. Both of them are targets of retinoid X receptor (RXR)/ LXR in macrophages [16,26]. Result presented in Figure 1A show that in CD4+ T cells, ATRA specifically induced RNA expression of ABCA1, while it has only minor effect on ABCG1 RNA expression. Similar regulation was also observed in macrophages [19]. The mechanism of regulation of ABCA1 and ABCG1 expression could be potentially different. The expression of two other genes from the same subgroup-ABCA3 and ABCG4 were also tested for specificity. None of their expressions changed in response to ATRA treatment (Figure 1A).

### Increased ABCA1 gene expression parallels with elevated cellular cholesterol efflux

**Figure 2:**
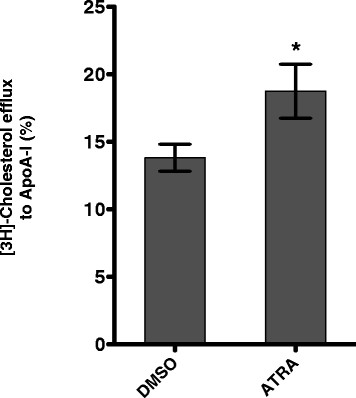
**ATRA stimulates ABCA1-mediated cholesterol efflux.** Activated CD4+ T cells were treated with DMSO or 5 μM ATRA and loaded with radioactive cholesterol for 24 hours and cholesterol efflux to apo-A1 was estimated after 4 hours incubation in cholesterol efflux medium. The result is presented as mean ± the standard deviation (SD; n = 3). * p < 0.05 versus cells treated with DMSO.

ABCA1 plays an essential role in controlling cellular cholesterol level by mediating cellular free cholesterol efflux to lipid-free apo-A1 [10]. To determine whether ABCA1-mediated cholesterol efflux increased in response to ATRA treatment, anti-CD3/CD28 antibody primed CD4+ T cells were incubated in the absence or presence of ATRA. Cells were then labeled with [3 H]-cholesterol and free cholesterol efflux to Apo-A1 was determined (Figure 2). As expected, cholesterol efflux to Apo-A1 increased in response to ATRA treatment by about 40%. The increase in cholesterol efflux parallels the induction of ABCA1 expression indicating that the increased cholesterol efflux is mediated by ABCA1.

### Retinoic acid and LXR ligand – TO-901317 have synergistic effects on ABCA1 expression and cholesterol efflux

**Figure 3:**
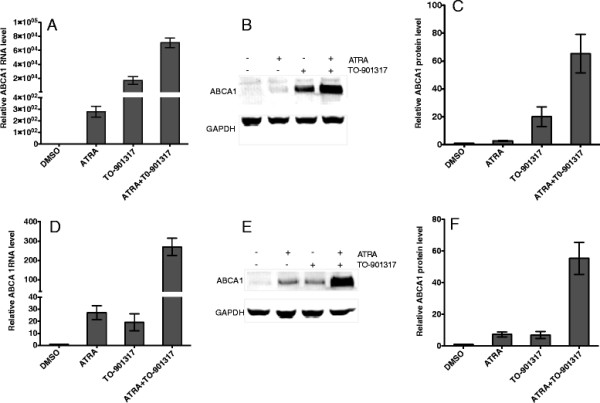
**ATRA and TO-901317 synergistically induce ABCA1 expression.A**) Primed human CD4+ T cells were incubated with 1 μM of ATRA and 1 μM of LXR-agonist TO-901317 for 2 days. ABCA1 mRNA was measured and normalized to the level of GAPDH mRNA and relative RNA fold changes compared to that from DMSO treated cells were plotted. Results are representative of three independent experiments. Data are presented as mean ± the standard deviation. **B**) ABCA1 and GAPDH proteins were detected as described in Methods. **C**) The intensity of the ABCA1 protein bands in (**B**) was quantified and normalized to that of GAPDH. The relative intensity compared to DMSO treatment was plotted. Results are representative of three independent experiments. Data are presented as mean ± the standard deviation. **D**) and **E**) The synergistic effects of 5 μM of ATRA and 1 μM of TO-901317 on ABCA1 RNA and protein expression, respectively, in Jurkat cells. **F**) The intensity of the bands representing ABCA1 protein in (**E**) was quantified and normalized to that of GAPDH protein. The relative intensity compared to DMSO treatment was plotted.

ABCA1 is regulated mainly at the transcription level. LXR and RXR, and their ligands are the most potential activators for ABCA1 expression and lipid efflux [20]. They up-regulate ABCA1 mRNA expression in a wide range of cells including macrophages, neuronal and intestine cells [19,27-32]. RXR forms heterodimers with RAR or LXR and binds to the direct repeats separated by 4 nucleotides (DR4) on the proximal promoter of ABCA1 and up-regulate ABCA1 gene expression. Combination of retinoic acid with natural oxysterols has been shown to have the most effect on ABCA1 levels [16,29,32,33]. It will be interesting to know whether ATRA and LXR agonist TO-901317 have synergistic effect on ABCA1 expression in T cells. As shown in Figure 3 A and B, ATRA or TO-901317 increased ABCA1 expression at RNA and protein level in primary CD4+ T cells, and the combination of ATRA and TO-901317 further enhanced ABCA1 expression. Treatment of Jurkat cells, a CD4+ T cell line, with ATRA and TO-901317 increased ABCA1 expression by 27- and 20-fold, respectively (Figure 3C) and treatment with both ATRA and TO-901317 increased ABCA1 expression by almost 300 fold showing synergistic effect of ATRA and TO-901317 on ABCA1 expression in T cells (Figure 3D).

**Figure 4:**
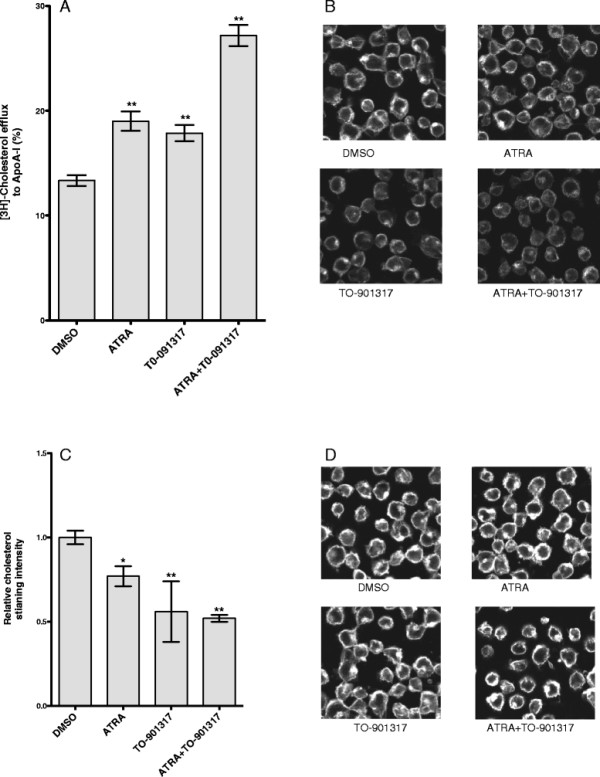
**ATRA and TO-901317 synergistically increase cellular cholesterol efflux. A) ATRA and TO-901317 increased ABCA1 dependent cholesterol efflux in Jurkat cells.** Jurkat cells were treated with 5 μM ATRA, or 5 μM TO-901317 or 5 μM each of ATRA and TO-901317 for 24 hours along with radioactive cholesterol and cholesterol efflux to apo-A1 was estimated after 24 hours of incubation in cholesterol efflux medium. The results are presented as mean ± the standard deviation (SD; n = 3). ** p < 0.01 versus cells treated with DMSO. **B-D**) Cholesterol staining with Filipin III. 1G5 cells treated with 5 μM of ATRA and 1 μM of TO-901317 for 3 days and then incubated without (**B**, **C**) or with (**D**) 60 μM of water soluble cholesterol for 30 minutes at 37 °C. Cells were then fixed and stained with Filipin III as described in Methods. Scale bar 5 μm. **C**) The intensity of cholesterol staining in (**B**) was analyzed as described in Methods. The relative intensity compared to DMSO treatment was plotted. Data represents three individual fields with 40–60 cells per field. *p < 0.05; **p < 0.01.

Next we investigated the synergistic effect of ATRA and TO-901317 on free cholesterol efflux to apo-A1 in Jurkat cell. By 48 hours, ATRA alone increased cholesterol efflux by 50%, whereas TO-901317 increased cholesterol efflux by 40%. Combination of ATRA and TO-901317 further increased cholesterol efflux by about 2-fold (Figure 4A). Parallel increases in ABCA1 level and cholesterol efflux strongly suggest that the cholesterol efflux is mediated by ABCA1.

Increased cholesterol efflux should lower cellular cholesterol level. To confirm this, we performed cellular cholesterol staining with Filipin III. Filipin III is a polyene antibiotic that interacts with cholesterol but not with cholesteryl esters. The staining was found mostly in the plasma membrane (Figure 4B). This is in line with the cholesterol concentration being higher in the plasma membrane than other cellular membranes. Some staining could also be detected in the endocytic-recycling compartment, a perinuclear compartment [34]. Upon ATRA treatment, the Filipin staining was reduced. Similar result was also observed in cells treated with TO-901317 (Figure 4B and 4C) and together, ATRA and TO-901317 reduced cholesterol staining by 50%. To replenish cholesterol to cells treated with ATRA and/or TO-901317, cells were incubated in the medium containing water-soluble cholesterol for 1 hr and stained with Filipin. Results in Figure 4 D shows that cholesterol could be replenished in cells treated with ATRA and or TO-901317.

Taken together, these results demonstrated that ATRA and TO-901317 have synergistic effect on reducing cellular cholesterol level in T cells by up-regulation of ABCA1 expression and ABCA1-dependent cholesterol efflux.

### ATRA and TO-901317 synergistically inhibit HIV-1 infection by reducing cellular cholesterol level

Cholesterol is important for a number of virus infections, including HIV-1 [2-5,35,36]**.** Cholesterol on both viral and cellular membrane is required for successful infection of HIV-1. Removal of cholesterol from HIV-1 with cholesterol extraction reagent ß-cyclodextrin resulted in inactivation of the virus [8]. ATRA has inhibitory effect on HIV-1 infection [37-40]. However, the mechanism of ATRA induced HIV-1 inhibition has not been fully understood. We hypothesize that ATRA could inhibit HIV-1 infection by reducing the cellular cholesterol. To test this hypothesis, we first investigated the effect of ATRA on HIV-1 infection using 1G5 cells, a reporter Jurkat cell line with integrated luciferase gene under the control of HIV-1 LTR promoter. 1G5 cells have low basal level of luciferase expression and could be activated by HIV-1 infection or by HIV-1 *tat*[41]. This cell line has been used to measure the HIV replication since a good correlation has been shown between the level of viral replication and the level of luciferase activity.

**Figure 5:**
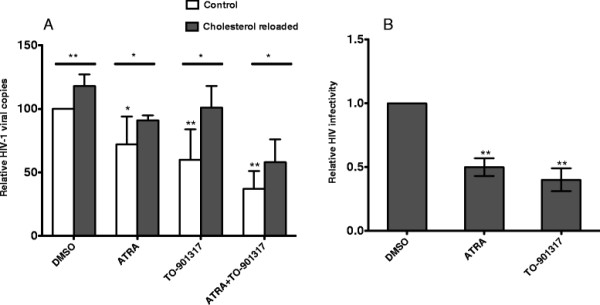
**ATRA and TO-901317 inhibit HIV-1 entry and replication. A) 1G5 cells were incubated for 3 days in the presence of ATRA or TO-901317.** Untreated cells (open bar) or cells treated with reconstituted water-soluble cholesterol (close bar), were then infected with HIV-1 for one hour, lysed and the amount of viral DNA was quantified by realtime PCR using R/U5 primer as described in Methods. The relative fold change of viral DNA compared to that from DMSO treatment was plotted. The results represent four independent experiments. Data are presented as mean ± the standard deviation. *p < 0.05; ** p < 0.01. **B**) 1G5 cells were pretreated with ATRA or TO-901317 for one day, and then infected with HIV-1 and were cultured in presence of ATRA and TO-901317. Luciferase activity was determined on 2 and 4 days after infection. The infectivity of HIV-1 was calculated by the relative fold change of luciferase on day 4 compared to that of basal level on day 2. The relative fold change compared to that of DMSO treatment was plotted. The experiment was performed in triplicates and the result is representative of two independent experiments. Data are presented as mean ± the standard deviation. ** p < 0.01 versus cells treated with DMSO.

ATRA and TO-901317 up-regulated ABCA1 expression and decreased cellular cholesterol in Jurkat cells and in 1G5 cells to the similar level (data not shown). To test the inhibitory effect of ATRA on HIV-1 infection, 1G5 cells were cultured in the presence and absence of ATRA for 3 days and infected with HIV-1. One hour after virus infection, virus entry was detected by quantitative PCR using primers that detect the accumulation of early R/U5 viral DNA from reverse transcription [42]. Compared with control, entry of HIV-1 virus into ATRA treated cells was reduced by 30% (Figure 5A). Cholesterol replenishment to ATRA treated cells reversed the inhibitory effect on HIV-1 entry indicating that the inhibition of HIV-1 entry was due to decreased cellular cholesterol level. Similar effect was also observed in cells treated with LXR agonist-TO-901317. The inhibitory effect of TO-901319 on HIV-1 virus entry is consistent with earlier findings [7,43]. Since ATRA and TO-901317 have synergistic effect on ABCA1 expression and cholesterol traffic, we hypothesized that ATRA and TO-901317 could reduce HIV-1 entry synergistically. As expected, virus entry in 1G5 cells treated with both ATRA and TO-901317 was reduced by 67%.

Next we tested whether ATRA can inhibit HIV-1 replication in 1G5 cells. The level of luciferase activity in these cells driven by HIV-1 LTR is proportional to viral entry, integration, and transcriptional activity. 1G5 cells were pretreated with ATRA for one day, and then infected with HIV-1. Infected cells were continuously cultured in the presence of ATRA for 4 days. Four days after HIV-1 infection, luciferase activity increased by more than 10 times compared to uninfected cells (data not shown), indicating successful virus infection. The HIV-1 replication level was calculated by the fold change of luciferase activity after 4 days of infection to the basal luciferase activity after 2 days of infection. Compared to vehicle treatment, HIV-1 infectivity was reduced by 50% and 60%, respectively, in cells treated with ATRA and TO-901317 (Figure 5A). Cell growth and viability were not affected under these conditions (data not shown). Based on these data we conclude that ATRA could inhibit HIV-1 infection by reducing virus entry and combination of ATRA and TO-901317 has the most inhibitory effect.

## Discussion

Vitamin A plays critical roles in T cell development and functions. Retinoic acid (RA) and ATRA, metabolites of vitamin A have been shown to be involved in multiple T cell effector responses through their binding to RAR, a ligand-activated transcription factor [21,22]. Antigen presenting cells provide the RA to the antigen primed T cells and promote the development of Treg cells [44]. Recent data show that RA is involved in development of both T helper and Treg cells [21,22]. ATRA up-regulates ABCA1 expression only in activated CD4+ T cells (Figure 1E), indicating that induction of ABCA1 by ATRA may play an important role in immune response.

Cellular cholesterol is a component of the plasma membrane and is also essential in cell proliferation. Regulation of intracellular cholesterol levels has been proposed as a mechanism to regulate T cell proliferation [45]. Intracellular cholesterol level is regulated by two competing pathways, cholesterol uptake and efflux, and ABCA1 plays a major role in the cholesterol efflux pathway. In this study, we demonstrated that ATRA induces ABCA1 expression and ABCA1-dependent cholesterol efflux in activated primary human CD4+ T cells and Jurkat cells implying that RA could affect T cell functions by regulating the cellular cholesterol levels.

ATRA is known to induce ABCA1 expression in macrophages either by RAR/RXR pathway or through induction of LXR and LXR/RXR pathway [20]. In the RAR/RXR pathway, ATRA was shown to up-regulate ABCA1 gene expression through direct binding of RAR/RXR heterodimers to the DR4 element in the ABCA1 promoter [13]. In the alternate pathway, ATRA up-regulates LXR, and LXR/RXR heterodimer induces ABCA1 expression through its interaction with the ABCA1 promoter [28,46]. Activation of T cells is known to up regulate LXR level [47], leading to the possibility that the synergistic effect of ATRA and LXR agonist is due to the activation of ABCA1 promoter by both RXR/RAR and RXR/LXR heterodimers in activated T cells.

Cholesterol is an essential component of cell membrane as well as viral membrane. The essential role of cholesterol in different steps of viral infection and replication has been demonstrated for a number of viruses [35,36]. Virus entry is one critical step requiring cholesterol. For HIV-1, cholesterol in both viral and target cell membrane is required for successful viral infection [8,48,49]. Depletion of cholesterol using cyclodextrin compounds has been demonstrated to have significant inhibitory effect on HIV-1viral infectivity in vitro [8]. ABCA1 mediated cholesterol efflux has been shown to inhibit HIV-1 infection in macrophages [6,7]. Our results show that ATRA mediated induction of ABCA1 and cholesterol efflux in activated T cells leads to the inhibition of HIV-1 infection. Both the induction of ABCA1 and the cholesterol efflux were further enhanced by LXR agonist similar to the data shown for macrophages [16,29,32,33].

ATRA is known to affect HIV-1 replication [37-40]. RA has been shown to inhibit HIV-1 production in stimulated T cell lines [50]. RA inhibited HIV-1 LTR activity and viral production in monocytes [39,51], and vitamin A deficiency enhanced the HIV-1 expression in rat model system [52]. Mechanism of RA mediated inhibition of HIV-1 replication is not known. Results presented here show that ATRA reduced the HIV-1 entry into CD4+ T cells by ABCA1 mediated cholesterol efflux and cholesterol replenishment abolished the inhibitory effect of ATRA (Figure 5B) strongly indicating that ABCA1 might play a role in this inhibition.

Growing attention has been drawn to dietary and plant-derived compounds targeting cholesterol and lipid rafts [53]. Retinoic acids, the bioactive metabolites of vitamin A, are likely candidates for natural repressors of HIV-1 in vivo. Vitamin A deficient diet can result in increased T cell pro-inflammatory responses and HIV-1 expression in HIV-1 transgenic rat [52]. Many HIV-1-induced diseases, including morbidity, mortality, and the rate of mother-to-child transmission, are inversely correlated with serum vitamin A levels [54,55]. Vitamin A supplementation has been shown to reduce HIV-1-associated disease and to slow the progression toward AIDS [56,57]. Additionally, retinoic acids appear to be useful as an adjuvant during vaccination to increase memory T cell responses and protection from viral infection at mucosal sites and it may facilitate the development of more effective vaccines against pathogens transmitted through mucus like HIV [58].

## Conclusions

In summary, results presented in this report demonstrated that ATRA specifically up regulated ABCA1 expression in CD4+ T cells. ATRA and LXR-agonist TO-901317 have synergistic effect on the induction of ABCA1 expression as well as anti-HIV-1 infection in CD4+ T cells. Taken together, retinoic acids along with LXR agonists could be potential candidates for systemic HIV-1 treatment.

## Methods

### Cells culture

Primary human CD4+ T cells were isolated from the peripheral blood mononuclear cells (PBMCs) of healthy donors using Dynabeads Untouched Human CD4+ T cells isolation kit (Invitrogen) following the manufacturer's instruction. Cells were cultured in RPMI 1640 supplemented with 10% dialyzed FBS, 100 U/ml penicillin, 100 μg/ml streptomycin, 2 mM L-glutamine, 50 U/ml IL-2 (PeproTech). To activate CD4+ T cells, cells were primed with anti-CD3 and anti-CD28 antibodies using Dynabeads CD3/CD28 T cell expander (Invitrogen). Jurkat E6.1 cell line, a CD4+ human T cell lymphoblast-like cell line, was purchased from ATCC. The 1G5 cell line, a clonal line derived from Jurkat cells stably transfected with an LTR (HIV)-luciferase construct was provided by the AIDS Research and Reference Reagent Program, Division of AIDS, National Institute of Allergy and Infectious Diseases, National Institutes of Health [41]. Jurkat cell lines were cultured as described [59]

### Reagents

ATRA, LXR agonist TO-901317, water-soluble cholesterol, phorbol myristate acetate (PMA), phytohemagglutinin (PHA), and Filipin III were purchased from Sigma-Aldrich (St. Louis, MO). Antibodies against ABCA1 and glyceraldehyde 3-phosphate dehydrogenase (GAPDH) were purchased from Abcam (Cambridge, MA).

### Reverse transcription and Realtime PCR

Total cellular RNA was extracted using RNAqueous®-4PCR Kit (Invitrogen). To quantitatively analyze gene expression, 200 ng of total RNA was used to synthesize the first strand DNA with random primers [59]. The real-time PCR was performed by using SYBR Green Master Mix (Qiagen) and the following primers: Abca1-forward 5’-GGTAGGAGAAAGAGACGCAAACAC-3’ and reverse 5'- AACAAGCCATGTTCCCTCAGC -3'; Abca3- forward 5'- GAGCATCTGAAAGACGCACTGC-3'and reverse 5’ ATCTCTGAAGGACGCTGCCACAAG-3; Abcg1-forward 5’– CCGACCGACGAC ACAGAGA -3’and reverse 5' – GCACGAGACACCCACAAACC -3’; Abcg4- forward 5' – GAGCCAGGGTCAGTGCATCT -3’, reverse 5' – GCAAGCCGAGTCCCTTAGA -3’. The quantities of ABCs mRNAs were normalized by the levels of GAPDH mRNA.

### Western blot for ABCA1

Whole cell proteins were extracted using M-PER mammalian protein extraction reagent with protease inhibitor cocktails (Thermo Scientific). Protein extracts were electrophoresed in a 4-12% gradient NuPAGE Bis Tris Gel (Invitrogen Life Technologies), and transferred to PVDF membrane and detected with fluorophore-labeled secondary antibody using Odyssey Infrared Imaging System (LI-COR Biotechnology).

### Cholesterol efflux assay

The assay was performed as described by Costet et al. [19]. Briefly cells were cholesterol loaded and radiolabeled for 24 hours in RPMI-1640 medium containing 0.2% bovine serum albumin (BSA), 50 ug/ml of acetylated low-density lipoprotein (Biomedical Technologies) and 1 μCi/ml of [3 H] cholesterol (Perkin Elmer) in the presence or absence of ATRA or TO-901317. Cells were washed with PBS, equilibrated for 30 min in RPMI-1640 medium with 0.2% BSA, and then incubated in cholesterol efflux medium (RPMI-1640 with 0.2% BSA, 5 ug/ml of purified apoA-1 (EMD Millipore)). For Cholesterol efflux analysis the samples were collected at 4 hours of incubation and radioactivity in the medium and cell lysate was counted by liquid scintillation counting. Cholesterol efflux was calculated as the percentage of the radioactivity recovered in the medium over the total radioactivity (cells plus media). Cholesterol efflux assay was performed in triplicates.

### Cholesterol replenishment and staining

To replenish cholesterol, Jurkat cells were incubated with cholesterol-saturated methyl-β-cyclodextrin at a concentration of 60 μM cholesterol for 60 minutes at 37 °C and then washed five times with PBS before being used in cholesterol staining and virus infection.

For cholesterol staining cells were allowed to rest in 0.01% poly-l-lysine–coated 8-well chamber slide for 5 min before a short spin, fixed with 3% formaldehyde (Thermo Scientific) for 1 hr at room temperature, washed with PBS, and incubated with freshly prepared Filipin III solution (250 μg/ml in PBS with 1% BSA) for 1 hr. Then, cells were washed with PBS and mounted in ProLong Anti-fade mounting media (Invitrogen) and were observed under an inverted two futon fluorescence microscope (LSM 710, Zeiss) at 720/460 nm with a 60X immersion lens. Images were acquired and analyzed using LSM 5 image browser. To measure Filipin III intensity, the total pixel intensity for same number of cells was recorded after subtracting background using Medical Image Processing, Analysis, and Visualization application. Forty to sixty cells were analyzed per view and three independent views were performed for each treatment.

### HIV-1 infection

1G5 cells were treated with ATRA or TO-901317 for 24 hours and infected with HIV-1 (10 ng of p24/10^6 cells) by spinoculation at 1200 g for two hours. Cells were washed extensively and incubated for four days in the presence of ATRA or TO-901317. Cells were harvested and the luciferase activity was measured using Luciferase Assay System [60]. To test HIV-1 virus entry**,** 1G5 cells were treated with ATRA and TO-901317 for three days and were reloaded with or without water-soluble cholesterol and infected with HIV-1 (10 ng of p24/10^6 cells). After additional one hour of incubation, cells were harvested and early viral DNA was measured by real-time PCR as described by Popik et al. [42].

### Statistical analysis

Statistical analysis was assessed by Student**’**s t test. A value of p < 0.05 was considered significant.

## Competing interests

The authors declare no conflict of interests.

## Authors’ contributions

JH designed the study and carried out ABCA1 RNA and protein estimations, cholesterol staining and analyzed the data and wrote the manuscript. YB carried out cholesterol efflux and viral infectivity assays. JY and RA participated in coordination and bioinformatic analysis of the gene array studies. AH carried out RTPCR. VN conceived and designed the study, analyzed the data and wrote the manuscript. All authors read and approved the final manuscript.
